# Mother’s physical activity during pregnancy and newborn’s brain cortical development

**DOI:** 10.3389/fnhum.2022.943341

**Published:** 2022-09-06

**Authors:** Xiaoxu Na, Rajikha Raja, Natalie E. Phelan, Marinna R. Tadros, Alexandra Moore, Zhengwang Wu, Li Wang, Gang Li, Charles M. Glasier, Raghu R. Ramakrishnaiah, Aline Andres, Xiawei Ou

**Affiliations:** ^1^Department of Radiology, University of Arkansas for Medical Sciences, Little Rock, AR, United States; ^2^Arkansas Children’s Nutrition Center, Little Rock, AR, United States; ^3^Arkansas Children’s Research Institute, Little Rock, AR, United States; ^4^College of Medicine, University of Arkansas for Medical Sciences, Little Rock, AR, United States; ^5^Department of Radiology and Biomedical Research Imaging Center, University of North Carolina at Chapel Hill, Chapel Hill, NC, United States; ^6^Department of Pediatrics, University of Arkansas for Medical Sciences, Little Rock, AR, United States

**Keywords:** physical activity during pregnancy, exercise in pregnant women, neonatal brain development, structural MRI, cortical thickness

## Abstract

**Background:**

Physical activity is known to improve mental health, and is regarded as safe and desirable for uncomplicated pregnancy. In this novel study, we aim to evaluate whether there are associations between maternal physical activity during pregnancy and neonatal brain cortical development.

**Methods:**

Forty-four mother/newborn dyads were included in this longitudinal study. Healthy pregnant women were recruited and their physical activity throughout pregnancy were documented using accelerometers worn for 3–7 days for each of the 6 time points at 4–10, ∼12, ∼18, ∼24, ∼30, and ∼36 weeks of pregnancy. Average daily total steps and daily total activity count as well as daily minutes spent in sedentary/light/moderate/vigorous activity modes were extracted from the accelerometers for each time point. At ∼2 weeks of postnatal age, their newborns underwent an MRI examination of the brain without sedation, and 3D T1-weighted brain structural images were post-processed by the iBEAT2.0 software utilizing advanced deep learning approaches. Cortical surface maps were reconstructed from the segmented brain images and parcellated to 34 regions in each brain hemisphere, and mean cortical thickness for each region was computed for partial correlation analyses with physical activity measures, with appropriate multiple comparison corrections and potential confounders controlled.

**Results:**

At 4–10 weeks of pregnancy, mother’s daily total activity count positively correlated (FDR corrected *P* ≤ 0.05) with newborn’s cortical thickness in the left caudal middle frontal gyrus (rho = 0.48, *P* = 0.04), right medial orbital frontal gyrus (rho = 0.48, *P* = 0.04), and right transverse temporal gyrus (rho = 0.48, *P* = 0.04); mother’s daily time in moderate activity mode positively correlated with newborn’s cortical thickness in the right transverse temporal gyrus (rho = 0.53, *P* = 0.03). At ∼24 weeks of pregnancy, mother’s daily total activity count positively correlated (FDR corrected *P* ≤ 0.05) with newborn’s cortical thickness in the left (rho = 0.56, *P* = 0.02) and right isthmus cingulate gyrus (rho = 0.50, *P* = 0.05).

**Conclusion:**

We identified significant relationships between physical activity in healthy pregnant women during the 1st and 2nd trimester and brain cortical development in newborns. Higher maternal physical activity level is associated with greater neonatal brain cortical thickness, presumably indicating better cortical development.

## Introduction

Physical activity is an essential element of a healthy lifestyle, and the most recent evidence-based Committee Opinion from the American College of Obstetricians and Gynecologists Committee on Obstetric Practice suggests that “In the absence of obstetric or medical complications or contraindications, physical activity in pregnancy is safe and desirable, and pregnant women should be encouraged to continue or to initiate safe physical activities (ACOG, 2020).” Since physical activity is known to improve both physical and mental health, one interesting question is whether physical activity of pregnant women would also benefit the developing fetal brain and therefore impact neurodevelopmental outcomes of their children. Studies have shown that physical activity during uncomplicated pregnancy not only causes no harm to offspring neurodevelopment but also may in fact improve neurodevelopmental outcomes (Moyer et al., 2016; Niño Cruz et al., 2018). For example, higher maternal physical activity during pregnancy was positively associated with an increase in offspring vocabulary score at age 15 months (Jukic et al., 2013) and overall language development at age 2 years (Polañska et al., 2015). In addition, higher physical activity during pregnancy was associated with higher Battelle Development Inventory score (Domingues et al., 2014) and lower risk of abnormal Ages and Stages Questionnaire score at age 1 year (Nakahara et al., 2021), as well as higher Wechsler Preschool and Primary Scale of Intelligence score and overall language skills at age 5 years (Clapp, 1996). Despite these findings, it is still unclear what the underlying mechanisms are whereby maternal exercise or higher physical activity during pregnancy can lead to better offspring neurodevelopment. Our speculation is that physical activity during pregnancy promotes fetal brain development, which consequently has long-term impact on children’s neurodevelopmental outcomes. With the rapid advances in neonatal neuroimaging data acquisition and analysis, it is possible to sensitively evaluate the potential effects of physical activity during pregnancy on early brain development, e.g., at newborn period, which is a time point that all prenatal influences have completed while postnatal influences have not started yet.

We hypothesized that higher physical activity among healthy pregnant women without medical complications are associated with better cortical brain development of their newborns. To test this hypothesis, we utilized data from our study which involved prospectively recruiting healthy pregnant women at first trimester of pregnancy, documenting many of the environmental and lifestyle factors during pregnancy including their physical activity levels, and neuroimaging their newborn brain development using MRI and new imaging data analysis tools. We focused on the evaluation of cortical thickness, which is an sensitive indicator of brain development during infancy and childhood (Sowell et al., 2004; Remer et al., 2017; Wang et al., 2019) with important implications in long term cognitive/behavior outcomes (Zubiaurre-Elorza et al., 2012; Menary et al., 2013; Qi et al., 2019).

## Materials and methods

### Subjects

All study procedures were approved by the Institutional Review Board at the University of Arkansas for Medical Sciences, and all participants (pregnant women) provided written informed consents for themselves and their newborns to be included in this study. The study cohort is the same as that in a previous study of relationships between pregnant women’s BMI and newborn’s cortical brain development (Na et al., 2021). In this study, we obtained physical activity data throughout pregnancy and performed additional data analysis. Pregnant women on second parity and singleton pregnancy conceived without assisted fertility treatments and ≥ 21 years of age were recruited from a larger cohort (clinicaltrials.gov identifier: NCT01131117), in which those with pre-existing medical conditions such as diabetes mellitus, seizure disorder, and serious psychiatric disorders; drug abuse issues or alcohol use or smoking during pregnancy; sexually transmitted diseases; medical complications developed during pregnancy such as gestational diabetes and pre-eclampsia were excluded. In addition, neonates born pre-term (< 37 weeks of gestation) or with medical conditions or medications known to influence growth and development (such as any birth defect, brain malformation, or brain injury associated with delivery), or those unable to complete a brain MRI examination during natural sleep were also excluded. In total, 46 pregnant women were recruited from the larger cohort, 2 newborns met the exclusion criteria, and 44 mother/newborn dyads were included in this study.

All pregnant women had six study visits at 4–10, ∼12, ∼18, ∼24, ∼30, and ∼36 weeks of pregnancy, respectively, and had their physical activity measured at each time point by wearing an accelerometer (Actical, Philips Respironics Co., Inc., Bend, OR) for 3–7 days. The monitor was placed on the participant’s ankle on the non-dominant side and programmed to record movement activity beginning at 11:59 p.m. on a given day. For each time point, to be included in the analyses, each participant needed to record at least three valid days with at least 2 weekdays and 1 weekend day of accelerometer data (device was worn continuously through the day and night). Total number of steps, total activity count, and minutes in each work intensity (sedentary, light, moderate, vigorous, determined according to the manufacturer recommendations) per day were summed over the valid wear period and then divided by the total number of valid days worn to derive average daily values for each of these measurements.

In addition, self-reported socioeconomic status data including education and income of the pregnant women and spouses/partners were obtained. The pregnant women’s body mass index (BMI) during early pregnancy was calculated by height and weight measured at ∼12 weeks of pregnancy. Birth weight and length of the newborns were retrieved from medical records; while head circumference was measured at age ∼2 weeks during the MRI study visit. In total, 44 pregnant women and their newborns completed the experimental procedures and were included in this study. Table 1 summarizes the demographic information of the study participants.

**TABLE 1:** Demographic information of the study participants.

	Mean ± SD (or counts)	Range (if applicable)
Child sex (boys/girls)	23/21	
Child race*	32/7/1/4	
Birth weight (kg)	3.5 ± 0.5	[2.2, 4.6]
Birth length (cm)	50.6 ± 2.7	[43.2, 54.6]
Head circumferences at MRI (cm)	36.1 ± 1.1	[32.6, 37.8]
Gestational age at birth (days)	274.9 ± 6.7	[261, 285]
Postnatal age at MRI (days)	14.3 ± 1.6	[11, 19]
Postmenstrual age at MRI (days)	289.3 ± 6.5	[277, 300]
Maternal age at delivery (years)	29.4 ± 4.0	[22.1, 38.2]
Maternal BMI at ∼12 weeks (kg/m^2^)	26.2 ± 5.6	[18.3, 36.5]
Mother’s education*	0/3/26/15	
Father’s education*	3/34/7/0	
Mother’s income*	7/9/21/7	
Father’s income*	4/0/20/20	

Child race*: (White/African American/American Indian/others).

Education*: (NA/no college/some college/graduate degree).

Income*: (N/A/ ≤ $20k/$20–50k/ > $50k).

### MRI data acquisition

An MRI examination of the brain during natural sleep without sedation was performed at the Arkansas Children’s Hospital Department of Radiology for each newborn at ∼2 weeks of postnatal age. The MRI data acquisition protocol has been described previously (Na et al., 2021). Specifically, the newborns were fed 15–30 min prior to the scan, swaddled in warm sheets, bundled using an Infant Immobilizer, and transferred to the scanner room after falling asleep. A pulse oximeter probe was placed on a foot to monitor oxygen saturation and heart rate, and mini-muffs and a headset were placed over the ears to protect the newborns from the noise generated during the scan. The MRI examinations were performed on a 1.5 Tesla Achieva MRI scanner (Philips Healthcare, Best, the Netherlands) with 60 cm bore size, 33 mT/m gradient amplitude, and 100 mT/m/ms maximum slew rate. A pediatric 8-channel SENSE head coil was used. A conventional neonatal MRI protocol was used for neuroradiologists to screen subjects for incidental findings, while 3D T1-weighted images with 7.1 ms TR, 3.2 ms TE, 8° flip angle, and 1 mm × 1 mm × 1 mm resolution were used for brain structural imaging and subsequent cortical brain morphometry analysis, particularly, measurements of cortical thickness. Imaging quality control was done on the scanner by experienced MRI techs. Scans were repeated if considerable motion artifacts presented.

### MRI data processing

All MR images were reviewed by one neuroradiologist. No incidental findings needing medical attention were noted. The pre-processing steps of structural MR images for cortical reconstruction were identical as those reported in a recent publication (Na et al., 2021). Specifically, the 3D T1-weighted images were post-processed by the iBEAT (Infant Brain Extraction and Analysis Toolbox) V2.0 software (developed by the Developing Brain Computing lab and Baby Brain Mapping lab at the University of North Carolina at Chapel Hill). iBEAT V2.0 is a newer version of the previous iBEAT software, utilizing advanced deep learning approaches to process pediatric brain structural MRI data such as 3D T1-weighted and/or 3D T2-weighted images. It has demonstrated superior capability and accuracy in infant structural MRI data post-processing based on datasets from multiple sites with various scanners and protocols. For neonatal MRI, the contrast between different neonatal brain tissues in T1-weighted images is sufficient for iBEAT V2.0 to process (Li et al., 2014; Sun et al., 2021). Specifically, for this study, 3D T1-weighted images were corrected for inhomogeneity before skull stripping, followed by tissue segmentation to gray matter, white matter and cerebrospinal fluid (Wang et al., 2018). The segmented brain images were then separated to left/right hemisphere and consequent steps were performed including topology correction, cortical surfaces (inner and outer) reconstruction and cortical parcellation (Li et al., 2014, 2019), based on neonate/infant specific developmental parcellation maps (Li et al., 2015; Wang et al., 2019). The UNC neonatal cortical surface atlas (Li et al., 2015) was used to parcellate brain cortex in each hemisphere to 34 different brain regions, and the mean cortical thickness (defined as the closest distance from the white surface to the pial surface at each surface’s vertex) of each cortical region was calculated for each subject, and used for later statistical analyses.

### Statistical analysis

To evaluate the relationships between mother’s physical activity during different trimesters of pregnancy and neonatal cortical thickness in all brain regions, Spearman’s rank partial correlation tests were used for the statistical analyses. Sex was included in the analyses as a covariate due to reported sex differences in brain cortical development in children. Total brain cortical volume was also included as a covariate in the analyses to account for variations in individual brain size that may be associated with a number of factors such as birth weight/length, head circumference, and gestational age at birth as well as postnatal age at the time of MRI. Other parameters such as parental socioeconomic status including education and income were not included as covariates due to limited sample size; nevertheless, Spearman’s correlation tests between brain cortical thickness of each region and each socioeconomic status parameter were performed and no significant relationship was identified. These sensitivity analyses indicated that the exclusion may be justified. Finally, given the potential interaction between BMI and physical activity in the pregnant women and our previous findings of relationships between mother’s BMI and offspring’s brain development (Ou et al., 2015; Na et al., 2021), we also tested the statistical model with and without adding the pregnant women’s BMI as a covariate. All analyses were applied to each hemisphere separately, and effect sizes (rho values) and significance level (*P*-values) between variables of interest were calculated. Benjamini-Hochberg correction of the false discovery rate (FDR) correction was used for multiple-comparison correction at each time point associated with the 68 brain cortical regions (left and right hemisphere combined), not over different time points, and correlations between physical activity parameters and regional cortical thickness with FDR corrected *P*-values ≤ 0.05 were regarded as significant. All statistics analyses were done in Matlab software (Version R2018b, The MathWorks, Inc., Natick, MA).

## Results

**FIGURE 1:**
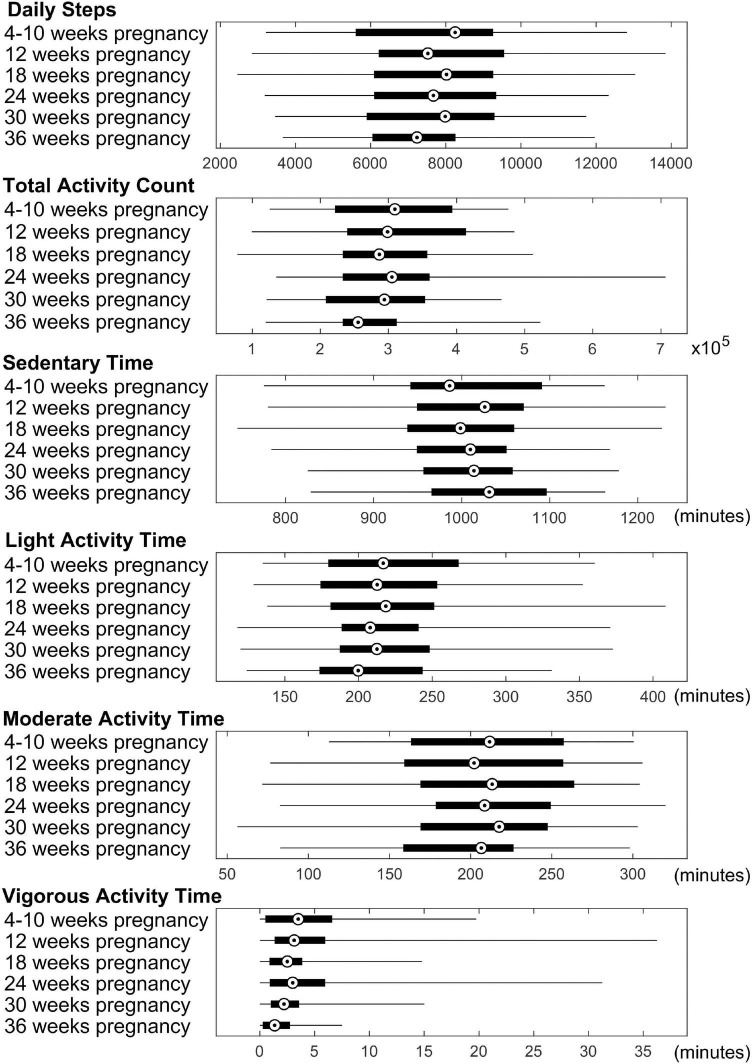
Summary of physical activity data for the pregnant women. Average daily steps, daily total activity count, and daily time spent in sedentary/light/moderate/vigorous activity modes throughout the pregnancy are presented. Median value, 25th and 75th percentiles, as well as range are shown for each measure.

In total, 44 pregnant women and their newborns (23 boys and 21 girls) completed the study and were included in this report. The demographic information of these participants are summarized in Table 1. Physical activity data of the pregnant women were downloaded from the Actical watches, and summary parameters including average of daily total number of steps, daily total activity count, daily total time in sedentary/light/moderate/vigorous activity modes for each time point throughout pregnancy (at 4–10, ∼12, ∼18, ∼24, ∼30, and ∼36 weeks, respectively) were illustrated in Figure 1. In general, daily step counts and daily total activity counts appeared to slightly decrease along pregnancy, and daily time spent in sedentary mode appeared to slightly increase along pregnancy, while daily time spent in light/moderate/vigorous activity modes appeared to slightly decrease along pregnancy. Nevertheless, ANOVA tests showed that none of these changes was significant. Additionally, the correlations between each physical activity parameter for each time point were represented in Supplementary Table 1.

**FIGURE 2:**
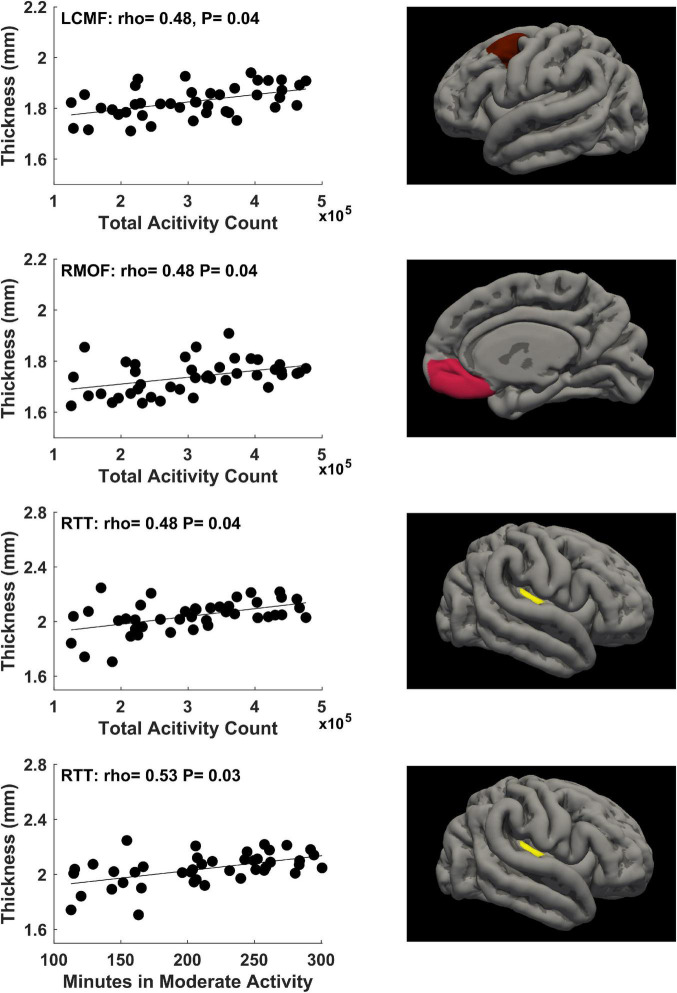
Significant positive correlations (*P* ≤ 0.05, FDR corrected) between mother’s physical activity at 4–10 weeks of pregnancy and newborn’s brain cortical thickness in the left caudal middle frontal (LCMF) gyrus, right medial orbital frontal (RMOF) gyrus, and right transverse temporal (RTT) gyrus.

For physical activity during the first trimester of pregnancy, significant correlations (*P* ≤ 0.05, FDR corrected) between mother’s physical activity at 4–10 weeks of pregnancy and newborn’s cortical thickness measures in 3 brain regions were identified (Figure 2). Specifically, mother’s average daily total activity count positively correlated with newborn’s cortical thickness in the left caudal middle frontal gyrus (rho = 0.48, *P* = 0.04), right medial orbital frontal gyrus (rho = 0.48, *P* = 0.04), and right transverse temporal gyrus (rho = 0.48, *P* = 0.04); in addition, mother’s average daily time spent in moderate activity mode positively correlated with newborn’s cortical thickness in the right transverse temporal gyrus (rho = 0.53, *P* = 0.03).

**FIGURE 3:**
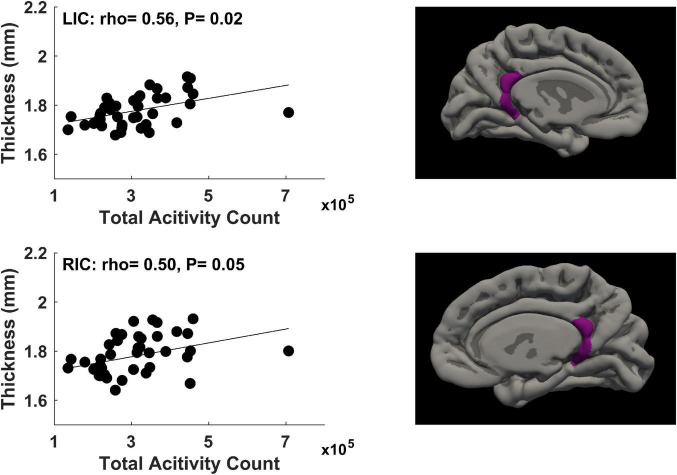
Significant positive correlations (*P* ≤ 0.05, FDR corrected) between mother’s physical activity at ∼24 weeks of pregnancy and newborn’s brain cortical thickness in the left isthmus cingulate (LIC) gyrus and right isthmus cingulate (RIC) gyrus.

For physical activity during the second trimester of pregnancy, significant correlations (*P* ≤ 0.05, FDR corrected) between mother’s physical activity at ∼24 weeks of pregnancy and newborn’s cortical thickness measures in 2 brain regions were identified (Figure 3). Specifically, mother’s average daily total activity count positively correlated with newborn’s cortical thickness in the left isthmus cingulate gyrus (rho = 0.56, *P* = 0.02) and right isthmus cingulate gyrus (rho = 0.50, *P* = 0.05).

For physical activity during the third trimester of pregnancy, there were no significant relationships (FDR corrected *P* ≤ 0.05) between mother’s physical activity and newborn’s brain cortical thickness.

### Exploratory analyses

To further investigate why significant relationships were identified in some time points but not in other time points during different trimesters of pregnancy, we investigated potential relationships with FDR corrected *P*-values in the range of 0.05–0.10 (Table 2). Additional correlations (0.05 < FDR Corrected *P* ≤ 0.10) were identified between mother’s physical activity during the first and second trimester of pregnancy and newborn’s cortical thickness. Specifically, at 4–10 weeks of pregnancy, average daily steps positively correlated with cortical thickness in 3 brain regions (left caudal middle frontal gyrus, right medial orbital frontal gyrus and right transverse temporal gyrus); average daily time spent in sedentary mode negatively correlated with cortical thickness in 2 brain regions (left pars triangularis gyrus and left transverse temporal gyrus); while average daily time spent in light/moderate/vigorous activity modes each positively correlated with cortical thickness in one brain region (left transverse temporal gyrus/left pars triangularis gyrus/medial orbital frontal gyrus). At ∼12 weeks of pregnancy, average daily total activity count positively correlated with cortical thickness in 9 brain regions (left caudal anterior cingulate gyrus, left caudal middle frontal gyrus, left isthmus cingulate gyrus, left parahippocampal gyrus, right isthmus cingulate gyrus, right medial orbital frontal gyrus, right parahippocampal gyrus, right frontal pole gyrus and right transverse temporal gyrus). At ∼ 18 weeks of pregnancy, average daily time spent in sedentary mode negatively correlated with cortical thickness in 1 brain region (right medial orbital frontal gyrus). At ∼24 weeks of pregnancy, average daily total activity count positively correlated with cortical thickness in 1 brain region (left parahippocampal gyrus), and average daily time spend in vigorous activity mode positively correlated with cortical thickness in 4 brain regions (left fusiform gyrus, left isthmus cingulate gyrus, left superior frontal gyrus and right isthmus cingulate gyrus). No other relationships were identified between mother’s physical activity at the first/second/third trimesters of pregnancy and newborn’s cortical thickness with FDR corrected *P*-values between 0.05 and 0.10.

**TABLE 2:** Additional relationships between mother’s physical activity and newborn’s brain cortical thickness at a level of FDR corrected *P*-values between 0.05 and 0.10.

Time points	Physical activity	Cortical region	Rho value	*P*-value (FDR corrected)
4–10 weeks	Daily steps	Caudal middle frontal (L)	0.45	0.09
	Daily steps	Medial orbital frontal (R)	0.46	0.09
	Daily steps	Transverse temporal (R)	0.47	0.09
	Sedentary time	Pars triangularis (L)	–0.47	0.08
	Sedentary time	Transverse temporal (L)	–0.48	0.08
	Light activity time	Transverse temporal (L)	0.50	0.07
	Moderate activity time	Pars triangularis (L)	0.46	0.09
	Vigorous activity time	Medial orbital frontal (L)	0.49	0.09
12 weeks	Total activity count	Caudal anterior cingulate (L)	0.40	0.10
	Total activity count	Caudal middle frontal (L)	0.41	0.10
	Total activity count	Isthmus cingulate (L)	0.52	0.06
	Total activity count	Parahippocampal (L)	0.43	0.10
	Total activity count	Isthmus cingulate (R)	0.41	0.10
	Total activity count	Medial orbital frontal (R)	0.43	0.10
	Total activity count	Parahippocampal (R)	0.47	0.10
	Total activity count	Frontal pole (R)	0.42	0.10
	Total activity count	Transverse temporal (R)	0.40	0.10
18 weeks	Sedentary time	Medial orbital frontal (R)	–0.50	0.10
24 weeks	Total activity count	Parahippocampal (L)	0.47	0.07
	Vigorous activity time	Fusiform (L)	0.49	0.06
	Vigorous activity time	Isthmus cingulate (L)	0.48	0.06
	Vigorous activity time	Superior frontal (L)	0.49	0.06
	Vigorous activity time	Isthmus cingulate (R)	0.45	0.08

To explore changes of the significant correlations illustrated in Figures 1, 2 throughout different time points during pregnancy, the rho values across these 6 time points for the physical activity/brain structural correlations were displayed in Supplementary Figures 1, 2. In general, the correlations were more prominent in the first two trimesters compared to the last trimester.

There were no significant correlations (all raw *P*-values were greater than 0.05) between maternal BMI and any physical activity parameters (average daily steps, total activity counts, times in sedentary/light/moderate/vigorous activity modes) measured at the 6 study visits during pregnancy. Nevertheless, as an exploratory measure to evaluate whether some of the associations we identified between maternal physical activity during pregnancy and newborn brain cortical development may still potentially be partially attributed to maternal body weight status, we included maternal BMI in the statistical model as an additional covariate and repeated the data analyses. Two of the significant correlations illustrated in Figures 1, 2 remained statistically significant (FDR corrected *P* ≤ 0.05). Specifically, daily time in moderate activity at 4–10 weeks of pregnancy positively correlated with cortical thickness in the right transverse temporal gyrus (rho = 0.52, FDR corrected *P* = 0.05), and daily total activity count at ∼24 weeks of pregnancy positively correlated with cortical thickness in the left isthmus cingulate gyrus (rho = 0.56, FDR corrected *P* = 0.02). The other four significant correlations illustrated in Figures 1, 2 now had FDR corrected *P*-values between 0.05 and 0.1, specifically, between daily total activity count at 4–10 weeks of pregnancy and cortical thickness in the left caudal middle frontal gyrus (rho = 0.46, FDR corrected *P* = 0.09), right medial orbital frontal gyrus (rho = 0.45, FDR corrected *P* = 0.09), and right transverse temporal gyrus (rho = 0.47, FDR corrected *P* = 0.09), and between daily total activity counts at ∼24 weeks of pregnancy and cortical thickness in the right isthmus cingulate gyrus (rho = 0.49, FDR corrected *P* = 0.07).

We further tested potential impact of parental social economic status that were excluded in the main results using variance inflation factor (VIF) analysis following a regression model. We established a linear regression model for all the six significant correlations, respectively. Adding more covariates such as parental social economic parameters increased the VIF of all variables slightly, indicating slightly higher multicollinearity of the regression model. In addition, the regression models also confirmed that all additional socioeconomic variables had a *p*-value > 0.05, indicating those variables did not have a significant impact in the regression.

## Discussion

In this study, we evaluated whether there are associations between maternal physical activity level during pregnancy and newborn’s brain cortical development. Our findings showed that during the first trimester (specifically, at 4–10 weeks of pregnancy) and second trimester (specifically, at ∼24 weeks of pregnancy), physical activity measures such as daily total activity count and daily time with moderate activity significantly correlated with cortical thickness in several brain regions. Furthermore, a number of potential correlations were also identified between cortical thickness in multiple brain regions and maternal physical activity measures such as daily steps, total activity count, sedentary and light/moderate/vigorous activity times at 4–10, ∼12, ∼18, or ∼24 weeks of pregnancy. All of the significant correlations (FDR corrected *P* ≤ 0.05) and potential correlations (0.05 < FDR corrected *P* ≤ 0.10) we identified suggested that higher maternal physical activity and less sedentary behavior during pregnancy is associated with greater cortical thickness and presumably better cortical development in newborns. Interestingly, we did not find significant relationships between newborn’s cortical brain development and mother’s physical activity level during late pregnancy. While limited sample size may be one of the reasons for this, it is also possible that physical activity during early pregnancy has a more prominent impact on fetal cortical brain development, as some literature has suggested that physical activity promotes neurogenesis in human and animal models (van Praag, 2008) which primarily occurs during early pregnancy (Stiles and Jernigan, 2010).

Relationships between exercise during pregnancy and offspring neurodevelopmental outcomes have been reported by several studies (Niño Cruz et al., 2018). For example, in a study involving a large cohort of children, maternal physical activity during pregnancy was positively associated with neurodevelopmental assessment scores at 12 months of age, after controlling for potential confounders (Domingues et al., 2014). In another study, leisure time physical activity of pregnant women positively correlated with child’s language development assessed at age 2 year using the Bayley Scales of Infant and Toddler Development (Polañska et al., 2015). Nevertheless, the underlying mechanisms of these types of reported relationships are unknown. Potential biological mechanisms such as placental transfer has been suggested (Robinson and Bucci, 2012). An animal study on rats investigated the effects of maternal physical activity on gene expression of the neurotrophic factors, and upregulation was found in placenta of the very active mothers (Fragoso et al., 2020). Additionally, other studies found voluntary exercise during pregnancy significantly increased the level of neurotrophic factors protein in hippocampus of rat pups; and female offspring born to pregnant mice with voluntary running wheels showed enhanced cell proliferation in the dorsal hippocampus (Akhavan et al., 2013; Yau et al., 2019). These animal studies suggest that maternal physical activity during pregnancy may promote fetal brain development in a biological point of view. Our speculation is that early changes in the brain may consequently have long-term impact on children’s neurodevelopmental outcomes. Our findings are consistent with the premise of our speculation, although longitudinal follow-up studies will be necessary to confirm the persistent effects of maternal physical activity during pregnancy on offspring brain development and the eventual changes in long-term neurodevelopment.

Our results also suggest that timing and intensity of the physical activity during pregnancy matter, consistent with literature findings (Clapp, 2003). Low/moderate-intensity maternal physical activity in early pregnancy were observed to have no harmful effect on the developing child and instead improve placental circulation; while high exercise volume in late pregnancy were not recommended (Bauer et al., 2020). Our findings of relationships between total activity counts as well as daily time in moderate activity in the first and second trimester of pregnant women and newborn’s brain cortical development may potentially inform important time window and specific strategies for potential intervention.

In our study, specific cortical regions we identified with significant relationships between maternal physical activity and cortical thickness included the caudal middle frontal gyrus, the medial orbital frontal gyrus, the transverse temporal gyrus, and the isthmus of cingulate gyrus. The structural development of these cortical regions play important roles in various functional domains. The middle frontal gyrus is involved in many brain functions including literacy, numeracy, and attention (Japee et al., 2015; Koyama et al., 2017), and the caudal part of the middle frontal gyrus is associated with tasks such as sentence/discourse generation (Bourguignon, 2014). The orbitofrontal cortex has been shown to play an important role in executive functioning, such as goal-directed behavior (Huang et al., 2020), reward (Kringelbach, 2005), working memory and decision making (Euston et al., 2012). The transverse temporal gyrus, also known as the Heschl’s gyrus, is part of the primary auditory cortex and is important for acoustic processing (Warrier et al., 2009). The cingulate cortex is part of the limbic system and is involved in emotion processing, behavior regulation, learning and memory (Rolls, 2019). While the potential associations between maternal physical activity during pregnancy and children’s outcomes in these functional domains still need more extensive research, and cannot be addressed in this report because of our focus on neonatal brain development instead of neurocognition or neurobehavior, our study provides important and novel findings on region-specific neonatal brain structural changes and may guide future longitudinal studies on brain function and neurodevelopmental outcomes associated with maternal physical activity.

Finally, there are extensive studies reporting relationships between exercise and associated brain structural and functional alterations in children and in adults (Ruscheweyh et al., 2011; Mandolesi et al., 2018; Szulc-Lerch et al., 2018; Cabral et al., 2019; Mora-Gonzalez et al., 2019; Zhang et al., 2021). For example, increase in physical activity was positively associated with increase in memory score and increase in cortical thickness of prefrontal and cingulate cortex (Ruscheweyh et al., 2011). In addition, exercise in long-term pediatric brain tumor survivors was associated with increase in cortical thickness in brain regions such as pre- and postcentral gyri, superior temporal gyrus, and parahippocampal gyrus (Szulc-Lerch et al., 2018). Furthermore, studies have also demonstrated associations between physical activity and cognitive function in children. For example, muscular strength, speed agility, and cardiorespiratory fitness were associated with executive function in children with overweight and obesity (Mora-Gonzalez et al., 2019), exercise can improve cognitive performance in children with and without ADHD (Christiansen et al., 2019), and children who engaged in physical activity demonstrated better executive functions in terms of inhibition and better planning abilities (Bidzan-Bluma and Lipowska, 2018). These studies linking exercise with changes in brain structures in frontal/temporal lobes and limbic systems and functions involving similar brain regions as we found between maternal physical activity and offspring brain development.

Despite the novelty of our study and data supporting potential benefits of increased physical activity in pregnant women on offspring brain development, our study has several limitations. The sample size is relatively small, which may have limited our ability to identify additional brain regions with significant correlations between mother’s physical activity and newborn’s cortical thickness. For the same reason, we had to take a conservative instead of aggressive approach in controlling for potential confounders, as adding too many covariates in our data analysis models may decrease the statistical power. We also reported results both with and without adding maternal BMI as a covariate in the statistical model, as our previous studies indicated that newborn’s brain development may also be associated with mother’s body weight status during pregnancy. Our results indicated that adding maternal BMI in the statistical model attenuated the relationships we identified between maternal physical activity and newborn cortical brain development, although it is not certain this was largely due to confounding/interaction of maternal BMI or due to decreased statistical power with additional covariate included in the statistical model. In addition, in this prospective and longitudinal study, while we have a relatively comprehensive monitoring of pregnant women’s physical activity by collecting data at multiple time points in all trimesters of pregnancy, it was not feasible to monitor physical activity for all time during pregnancy, and we only have one time point in offspring brain imaging. Whether changes in the developing brain associated with maternal physical activity are persistent in postnatal life and whether they can be mediated by postnatal factors are unknown.

## Conclusion

Utilizing longitudinal evaluation of physical activity in pregnant women throughout pregnancy and advanced neonatal neuroimaging data analyses, we identified significant relationships between physical activity in healthy pregnant women during the 1st and 2nd trimester and brain cortical development in newborns. Higher maternal physical activity level and less sedentary behavior is associated with greater neonatal brain cortical thickness, presumably indicating better cortical development. Our study provides the first direct evidence that physical activity during uncomplicated pregnancy may be beneficial for offspring brain development.

## Data availability statement

The raw data supporting the conclusions of this article will be made available by the authors, without undue reservation.

## Ethics statement

The studies involving human participants were reviewed and approved by the Institutional Review Board (IRB) at the University of Arkansas for Medical Sciences (UAMS). Written informed consent to participate in this study was provided by the participants’ legal guardian/next of kin.

## Author contributions

XO designed the study and obtained funding. XO and AA supervised data collection. XN, RR, ZW, LW, GL, CG, and RRR performed data analysis. XO, XN, NP, MT, AM, and AA drafted the manuscript. All authors edited the manuscript and provided final review.
