# Supplementary material for: Mother’s physical activity during pregnancy and newborn’s brain cortical development
Source: Front Hum Neurosci. 2022 Sep 6;16:943341. doi: 10.3389/fnhum.2022.943341 (PMC9486075; doi:10.3389/fnhum.2022.943341)
Supplement: Supplementary file 1 [file Data_Sheet_1.docx]

**Supplementary Materials**

Supplementary Figure 1. Effect sizes of Spearman’s correlation between mother’s physical activity of 6 time points during pregnancy and newborn’s brain cortical thickness in the left caudal middle frontal (LCMF) gyrus, right medial orbital frontal (RMOF) gyrus, and right transverse temporal (RTT) gyrus.

Supplementary Figure 2. Effect sizes of Spearman’s correlation between mother’s physical activity of 6 time points during pregnancy and newborn’s brain cortical thickness in the right transverse temporal (RTT) gyrus, left isthmus cingulate (LIC) gyrus and right isthmus cingulate (RIC) gyrus.

Supplementary Table 1. Correlation of average daily steps, daily total activity count, and daily time spent in sedentary/light/moderate/vigorous activity modes throughout the pregnancy of 6 time points respectively.

| **Physical Activity** | **Time Points** | **rho Value** |
| --- | --- | --- |
| Daily steps | 4-10 weeks vs. 12 weeks | 0.79* |
|  | 4-10 weeks vs. 18 weeks | 0.79* |
|  | 4-10 weeks vs. 24 weeks | 0.73* |
|  | 4-10 weeks vs. 30 weeks | 0.61* |
|  | 4-10 weeks vs. 36 weeks | 0.47 |
|  | 12 weeks vs. 18 weeks | 0.76* |
|  | 12 weeks vs. 24 weeks | 0.83* |
|  | 12 weeks vs. 30 weeks | 0.54* |
|  | 12 weeks vs. 36 weeks | 0.39 |
|  | 18 weeks vs. 24 weeks | 0.77* |
|  | 18 weeks vs. 30 weeks | 0.50* |
|  | 18 weeks vs. 36 weeks | 0.49 |
|  | 24 weeks vs. 30 weeks | 0.56* |
|  | 24 weeks vs. 36 weeks | 0.52 |
|  | 30 weeks vs. 36 weeks | 0.50 |
| Total activity count | 4-10 weeks vs. 12 weeks | 0.84* |
|  | 4-10 weeks vs. 18 weeks | 0.79* |
|  | 4-10 weeks vs. 24 weeks | 0.73* |
|  | 4-10 weeks vs. 30 weeks | 0.68* |
|  | 4-10 weeks vs. 36 weeks | 0.49 |
|  | 12 weeks vs. 18 weeks | 0.76* |
|  | 12 weeks vs. 24 weeks | 0.81* |
|  | 12 weeks vs. 30 weeks | 0.63* |
|  | 12 weeks vs. 36 weeks | 0.47 |
|  | 18 weeks vs. 24 weeks | 0.80* |
|  | 18 weeks vs. 30 weeks | 0.48* |
|  | 18 weeks vs. 36 weeks | 0.46 |
|  | 24 weeks vs. 30 weeks | 0.50* |
|  | 24 weeks vs. 36 weeks | 0.30 |
|  | 30 weeks vs. 36 weeks | 0.41 |
| Sedentary time | 4-10 weeks vs. 12 weeks | 0.81* |
|  | 4-10 weeks vs. 18 weeks | 0.78* |
|  | 4-10 weeks vs. 24 weeks | 0.74* |
|  | 4-10 weeks vs. 30 weeks | 0.61* |
|  | 4-10 weeks vs. 36 weeks | 0.60* |
|  | 12 weeks vs. 18 weeks | 0.79* |
|  | 12 weeks vs. 24 weeks | 0.72* |
|  | 12 weeks vs. 30 weeks | 0.59* |
|  | 12 weeks vs. 36 weeks | 0.51* |
|  | 18 weeks vs. 24 weeks | 0.80* |
|  | 18 weeks vs. 30 weeks | 0.49* |
|  | 18 weeks vs. 36 weeks | 0.58* |
|  | 24 weeks vs. 30 weeks | 0.52* |
|  | 24 weeks vs. 36 weeks | 0.59* |
|  | 30 weeks vs. 36 weeks | 0.62* |
| Light activity time | 4-10 weeks vs. 12 weeks | 0.74* |
|  | 4-10 weeks vs. 18 weeks | 0.73* |
|  | 4-10 weeks vs. 24 weeks | 0.77* |
|  | 4-10 weeks vs. 30 weeks | 0.71* |
|  | 4-10 weeks vs. 36 weeks | 0.59* |
|  | 12 weeks vs. 18 weeks | 0.69* |
|  | 12 weeks vs. 24 weeks | 0.68* |
|  | 12 weeks vs. 30 weeks | 0.72* |
|  | 12 weeks vs. 36 weeks | 0.54* |
|  | 18 weeks vs. 24 weeks | 0.81* |
|  | 18 weeks vs. 30 weeks | 0.63* |
|  | 18 weeks vs. 36 weeks | 0.53* |
|  | 24 weeks vs. 30 weeks | 0.68* |
|  | 24 weeks vs. 36 weeks | 0.67* |
|  | 30 weeks vs. 36 weeks | 0.56* |
| Moderate activity time | 4-10 weeks vs. 12 weeks | 0.83* |
|  | 4-10 weeks vs. 18 weeks | 0.81* |
|  | 4-10 weeks vs. 24 weeks | 0.64* |
|  | 4-10 weeks vs. 30 weeks | 0.63* |
|  | 4-10 weeks vs. 36 weeks | 0.62* |
|  | 12 weeks vs. 18 weeks | 0.81* |
|  | 12 weeks vs. 24 weeks | 0.64* |
|  | 12 weeks vs. 30 weeks | 0.55* |
|  | 12 weeks vs. 36 weeks | 0.56* |
|  | 18 weeks vs. 24 weeks | 0.67* |
|  | 18 weeks vs. 30 weeks | 0.46 |
|  | 18 weeks vs. 36 weeks | 0.55* |
|  | 24 weeks vs. 30 weeks | 0.33 |
|  | 24 weeks vs. 36 weeks | 0.52* |
|  | 30 weeks vs. 36 weeks | 0.54* |
| Vigorous activity time | 4-10 weeks vs. 12 weeks | 0.60* |
|  | 4-10 weeks vs. 18 weeks | 0.68* |
|  | 4-10 weeks vs. 24 weeks | 0.53* |
|  | 4-10 weeks vs. 30 weeks | 0.45 |
|  | 4-10 weeks vs. 36 weeks | 0.43 |
|  | 12 weeks vs. 18 weeks | 0.29 |
|  | 12 weeks vs. 24 weeks | 0.45 |
|  | 12 weeks vs. 30 weeks | 0.28 |
|  | 12 weeks vs. 36 weeks | 0.31 |
|  | 18 weeks vs. 24 weeks | 0.28 |
|  | 18 weeks vs. 30 weeks | 0.28 |
|  | 18 weeks vs. 36 weeks | 0.49 |
|  | 24 weeks vs. 30 weeks | 0.42 |
|  | 24 weeks vs. 36 weeks | 0.39 |
|  | 30 weeks vs. 36 weeks | 0.43 |

*The correlation survived the Bonferroni correction (corrected P ≤ 0.05 or raw P ≤ 0.003) for multiple comparison correction. The Bonferroni correction was performed within the 15 pairs of correlations for each activity parameter respectively.
